# Опыт успешного выполнения лапароскопической рукавной резекции желудка для лечения морбидного ожирения у пациентки с сольтеряющей формой врожденной дисфункции коры надпочечников

**DOI:** 10.14341/probl13206

**Published:** 2023-04-30

**Authors:** Е. А. Зацепина, В. С. Самойлов, А. П. Волынкина, А. В. Степаненко, Е. Е. Новичихина

**Affiliations:** Клиника «Город Здоровья» (Центр семейной медицины «Олимп Здоровья»); Клиника «Город Здоровья» (Центр семейной медицины «Олимп Здоровья»); Государственный научный центр Российской Федерации — Федеральный медицинский биофизический центр имени А.И. Бурназяна Федерального медико-биологического агентства России; Клиника «Город Здоровья» (Центр семейной медицины «Олимп Здоровья»); Воронежский государственный медицинский университет им. Н.Н. Бурденко; Клиника «Город Здоровья» (Центр семейной медицины «Олимп Здоровья»); Воронежский государственный медицинский университет им. Н.Н. Бурденко

**Keywords:** врожденная дисфункция коры надпочечников, морбидное ожирение, дефицит 21-гидроксилазы, глюкокортикостероиды, бариатрическая хирургия, лапароскопическая рукавная резекция желудка

## Abstract

В статье представлено клиническое наблюдение пациентки с сольтеряющей формой врожденной дисфункции коры надпочечников (ВДКН); (дефицит фермента 21-гидроксилазы (гомозиготная мутация I 172N)) в сочетании с морбидным ожирением, развившимся на фоне длительного приема высоких доз глюкокортикостероидов, которой была выполнена лапароскопическая рукавная резекция желудка. Особенностью представленного случая является устранение одной из причин декомпенсации заболевания, а именно избыточной массы тела, а также инсулинорезистентности, что требует приема больших доз глюкокортикоидов, в свою очередь, приводящего к ухудшению течения ожирения, вызывая тем самым порочный круг. Спустя 7 мес после оперативного лечения была достигнута цель — снижение дозы преднизолона на 25% при снижении массы тела на 72,1% от избыточной массы тела.

Представленный случай наглядно демонстрирует возможность выполнения бариатрической операции с целью лечения морбидного ожирения у пациентов с ВДКН при участии и контроле специализированной мультидисциплинарной команды. При наличии показаний к проведению бариатрического вмешательства ВДКН не должна являться абсолютным противопоказанием к подобным операциям. Соотношение профиля безопасности и профиля эффективности свидетельствует в пользу возможности выполнения бариатрических операций.

## Abstract (en)

The article presents a clinical observation of a patient with congenital adrenal dysfunction (CHD), a salt-losing form of 21-hydroxylase enzyme deficiency (homozygous mutation I 172N), and also with morbid obesity, due to long-term use of high doses of glucocorticosteroids, who underwent bariatric surgery — laparoscopic sleeve resection of the stomach. A feature of the presented case is the elimination of one of the causes of decompensation of the disease, namely, overweight, as well as insulin resistance, which requires the intake of large doses of glucocorticoids, which in turn leads to a worsening of the course of obesity, thereby causing a vicious circle. 7 months after surgical treatment, the goal was achieved — a reduction in the dose of Prednisolone by 25%, with a decrease in body weight by 72.1% of overweight.

The presented case clearly demonstrates the possibility of performing bariatric surgery for the treatment of morbid obesity in patients with CAH with the participation and control of a specialized multidisciplinary team. If there are indications for bariatric intervention, VDKN should not be an absolute contraindication to such operations, and the ratio of the safety profile and the efficacy profile testifies in favor of the need for their implementation.

## АКТУАЛЬНОСТЬ

Врожденная дисфункция коры надпочечников (ВДКН) — это группа аутосомно-рецессивных заболеваний, характеризующихся дефектом одного из ферментов или транспортных белков, принимающих участие в синтезе кортизола в коре надпочечников [1].

По последним данным, известно о 7 формах ВДКН, более 95% всех случаев связаны с дефицитом фермента 21-гидроксилазы [2]. При данной форме наблюдается дефицит кортизола, что по принципу отрицательной обратной связи в оси гипоталамус-гипофиз-надпочечники приводит к повышению уровня адренокортикотропного гормона (АКТГ) и, как следствие, к гиперплазии надпочечников (рис. 1). В результате стимуляции надпочечников происходит избыточное накопление стероидов, предшествующих ферментативному блоку [2][3].

**Figure:**
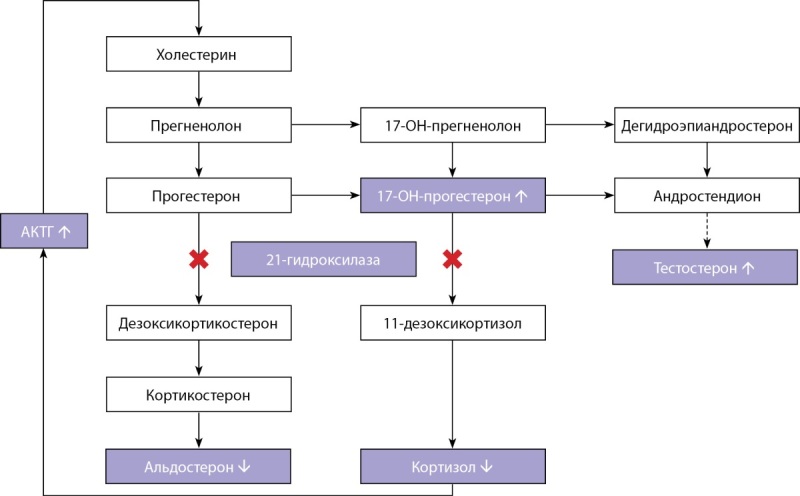
Рисунок 1. Патогенез ВДКН вследствие дефицита 21-гидроксилазы.Figure 1. Pathogenesis of CHD due to 21-hydroxylase deficiency.

Распространенность классических форм дефицита 21-гидроксилазы составляет от 1:14 000 до 1:18 000 живых новорожденных в мире. По данным неонатального скрининга в РФ, распространенность заболевания в отдельных регионах составляет от 1:5000 до 1:12 000, в целом по стране — 1:9638 живых новорожденных. Неклассическая форма данного дефицита встречается чаще — от 1:500 до 1:1000 среди общей популяции, а в некоторых изолированных этнических группах, характеризующихся высоким процентом близкородственных браков (например, евреи Ашкенази), распространенность может доходить до 1:50 до 1:100 [2][4][5].

Современные возможности скрининга новорожденных и вовремя начатая заместительная терапия глюкокортикостероидами (ГК) обеспечивают выживаемость детей с ВДКН. Неонатальный скрининг на 17-OH-прогестерон (17-ОНП) позволяет диагностировать классические формы дефицита 21-гидроксилазы. На втором этапе скрининга предпочтительно определение мультистероидного спектра методом тандемной масс-спектрометрии [6].

Целью лечения ВДКН является восполнение дефицита кортизола путем назначения адекватных доз ГК, а при необходимости — и альдостерона, на фоне параллельной супрессии гиперсекреции АКТГ. Сложности в подборе адекватной дозы ГК с соблюдением баланса между неэффективным подавлением продукции АКТГ и ятрогенным гиперкортицизмом приводят к различного рода нарушениям, развивающимся с первых месяцев жизни и прогрессирующим с течением лет, часто приобретая особую клиническую значимость. При недостаточной дозе ГК может наблюдаться отсутствие адекватного подавления АКТГ, что приводит к гиперандрогении и росту образований в ткани надпочечников. Терапия, направленная на нормализацию АКТГ, зачастую приводит к передозировке ГК и связанным с ней рискам и осложнениям, таким как низкий конечный рост, ожирение, нарушение минеральной плотности костной ткани, бесплодие, сердечно-сосудистые заболевания, а также снижение качества жизни [7][8]. Патогенез метаболических нарушений при классических формах дефицита 21-гидроксилазы представлен на рисунке 2.

**Figure:**
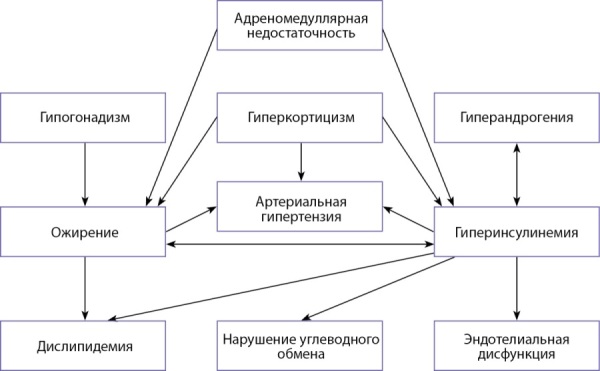
Рисунок 2. Патогенез метаболических нарушений при классических формах дефицита 21-гидроксилазы.Figure 2. Pathogenesis of metabolic disorders in classical forms of 21-hydroxylase deficiency

В исследовании Arlt W. и соавт. у 203 пациентов с ВДКН была подтверждена высокая распространенность метаболических нарушений, таких как ожирение (с частотой 41% случаев), избыточная масса тела (37%), гиперхолестеринемия (46%), инсулинорезистентность (29%), остеопения (40%) и остеопороз (7%) [7]. Схожие данные были получены и в российском исследовании 55 пациентов с классическими формами ВДКН в возрасте от 18 до 60 лет, в котором ИМТ≥25 кг/м2 встречался у 56% женщин и 47% мужчин, аналогично дислипидемия в 69 и 50%, артериальная гипертензия — в 10 и 14%, а нарушения углеводного обмена — в 17 и 7% случаев [8]. Современное понимание проблемы ожирения встречается все чаще и чаще и говорит о том, что развитие тяжелых (морбидных) форм ожирения у пациентов с ВДКН связано не только с приемом высоких доз ГК, но и с нарушениями пищевого поведения и, таким образом, носит смешанный характер, в том числе и алиментарный. Сложный комбинированный генез сопутствующей коморбидной патологии у данной группы пациентов требует особых подходов в терапевтической стратегии, а отсутствие адекватного лечения приводит к декомпенсации основного заболевания, что требует увеличения доз ГК, образуя порочный круг. Таким образом, значимое снижение веса в комплексном лечении пациентов с ВДКН и ожирением играет одну из ведущих ролей.

В настоящее время доказанно эффективным методом лечения ожирения, особенно морбидных форм, является бариатрическая хирургия. По ключевому механизму действия бариатрические вмешательства делят на преимущественно рестриктивные (рукавная резекция желудка) и преимущественно гипоабсорбтивные (одноанастомозное желудочное шунтирование, гастрошунтирование по Roux-en-Y и другие) [9].

Бариатрическая хирургия не только приводит к значительному снижению веса, но и позволяет длительно удерживать достигнутые результаты под регулярным наблюдением и контролем эндокринолога, а также при выполнении послеоперационных рекомендаций. В доступной современной зарубежной литературе описан лишь единичный опыт выполнения бариатрической операции у пациентки с ВДКН. В 2017 г. группа исследователей из Клинического центра Национального института здоровья, Бетесда, Мэриленд, США во главе с Ashwini Mallappa опубликовала положительные результаты выполнения лапароскопической рукавной резекции пациентке с морбидным ожирением (МО) и ВДКН. Авторы отметили, что на фоне значительного снижения веса после операции удалось достигнуть снижения дозы принимаемых ГК, а именно доза гидрокортизона была снижена на 34% через 15 мес после операции [10]. В отечественной литературе мы не встретили подобного опыта.

Вашему вниманию представлен клинический случай успешного выполнения лапароскопической рукавной резекции желудка у пациентки с ВДКН и МО, ассоциированным с приемом ГК, а также нарушениями пищевого поведения.

## ОПИСАНИЕ СЛУЧАЯ

Пациентка А., 31 год, с диагностированной на первом году жизни сольтеряющей формой ВДКН поступила в центр метаболической и бариатрической хирургии (г. Воронеж) с целью хирургического лечения морбидного ожирения.

При поступлении жалобы на избыточную массу тела, периодически отеки голеней, ощущение тяжести в нижних конечностях, периодические боли в позвоночнике, боль при движении в правом коленном суставе (в анамнезе повреждение крестообразной связки), одышку при интенсивной физической активности (подъем на 3 этаж), отсутствие менструаций в течение длительного периода, частый пищевой дискомфорт, связанный с погрешностями в питании, быструю утомляемость, психологический и физический дискомфорт, снижение качества жизни, связанные с избыточной массой тела.

Страдает ожирением с детства. Интенсивное увеличение массы тела в течение последних 4 лет. Течение заболевания прогрессирующее. Увеличение массы тела связывает с малоподвижным образом жизни, приемом ГК в связи с сопутствующей патологией (ВДКН), избыточным и нерегулярным питанием. По результатам анкетирования (Голландский опросник пищевого поведения DEBQ) у пациентки выявлен смешанный тип нарушения пищевого поведения с преобладанием эмоциогенного компонента. Также при подготовке к операции проведена оценка качества жизни с помощью опросника SF-36.

Максимальный вес 110 кг наблюдался на момент первичного обращения. Неоднократно предпринимала организованные попытки консервативного снижения массы тела, включая различные диеты, занятия спортом, психотерапию. Максимальный эффект — снижение веса на 10 кг с последующим рикошетным восстановлением массы тела с избытком. В предоперационном периоде проведена коррекция питания путем разработки индивидуальной гипокалорийной диеты под руководством бариатрического эндокринолога, диетолога. Удалось достичь снижения массы тела на 4 кг (3,6% исходной массы тела).

На момент оперативного лечения рост пациентки 157 см, масса тела 106 кг, ИМТ=43 кг/м2. «Идеальная» масса тела: 57 кг. Избыточная масса тела: 49 кг. Из анамнеза известно, что ВДКН выявлена при рождении на основании характерной клинической картины, подтверждена генетическим исследованием, в связи с чем сразу была начата заместительная терапия ГК. В 1994 и 2007 г. были выполнены операции феминизирующей пластики. В 2007 г. установлен диагноз: аменорея І, была назначена заместительная гормональная терапия Фемостоном 1/10 и 2/10 в дальнейшем без положительного эффекта. По данным УЗИ органов малого таза матка нормальных размеров. С 1994 г. по 2016 г. ежегодно наблюдалась и обследовалась на базе ФГБУ «НМИЦ эндокринологии». Были неоднократно зафиксированы значительные повышения уровней инсулина, индекса HOMA, 17-ОНП, тестостерона, прогестерона, андростендиона, пониженный уровень дигидроэпиандростерона сульфата (ДГЭА-С), лютеинизирующего гормона (ЛГ) ввиду нерегулярного приема пациенткой назначенных препаратов.

На этом фоне в 2008 г. отмечено развитие осложнений: по данным денситометрии установлены признаки остеопении, при МСКТ выявлена вторичная аденома дистального отдела латеральной ножки правого надпочечника размерами 3,5×3,0×2,8 см. При динамическом наблюдении в 2010 г. зарегистрировано увеличение размеров образования (3,92×3,2×3,1 см), а также появление еще двух образований в толще правого надпочечника и 4 образований в теле левого надпочечника размерами от 18×16×18 мм до 42×29×39 мм. В связи с наличием объемных образований обоих надпочечников высокой нативной плотности (до 20HU) с наличием зон некроза в левом надпочечнике, отрицательной динамикой размеров образований, сложностью достижения компенсации ВДКН, требующей назначения высоких доз ГК, сопровождающейся выраженной прибавкой массы тела, рассматривался вопрос о проведении двусторонней адреналэктомии. Учитывая индивидуальные высокие риски, принято решение воздержаться от проведения одномоментной двусторонней адреналэктомии, так как развитие абсолютной надпочечниковой недостаточности в условиях отказа от приема заместительной терапии ГК неминуемо приведет к летальному исходу. В 2010 г. была выполнена левосторонняя адреналэктомия, назначена заместительная гормональная терапия — преднизолон 5 мг 2 р/сут, флудрокортизон 0,1 мг ½ таб. утром, ¼ таб. в обед. С 2013 г. до момента госпитализации получала преднизолон 5 мг 2 р/сут, флудрокортизон 0,1 мг 1 р/сут.

Предоперационный период реализован при участии специализированной мультидисциплинарной команды, включающей бариатрического хирурга, бариатрического эндокринолога, диетолога, анестезиолога, совместно согласована персональная стратегия лечения. Предоперационно пациентка была переведена на парентеральную форму ГК — преднизолон 0,5 мл 2 раза в сутки в/м. Обследование по ВДКН проведено на догоспитальном этапе.

Пациентке установлен клинический диагноз: Морбидное ожирение. Врожденная дисфункция коры надпочечников, дефицит 21-гидроксилазы, сольтеряющая форма (гомозиготная мутация I 172N). Вторичные аденомы правого надпочечника, состояние после левосторонней адреналэктомии в 2010 г. Аменорея I. НАЖБП. Хронический гастродуоденит, вне обострения. Остеопения.

10.03.2022 выполнена лапароскопическая рукавная резекция желудка (рис. 3) по стандартной методике. После мобилизации большой кривизны желудка от уровня привратника и до угла Гиса в проксимальном направлении произведена аппаратная продольная резекция желудка на зонде 39Fr с использованием 4 60-мм картриджей. Далее линия резекции укрыта непрерывным погружным швом на всем протяжении. Операционное время составило 65 минут, кровопотеря 10 мл.

**Figure:**
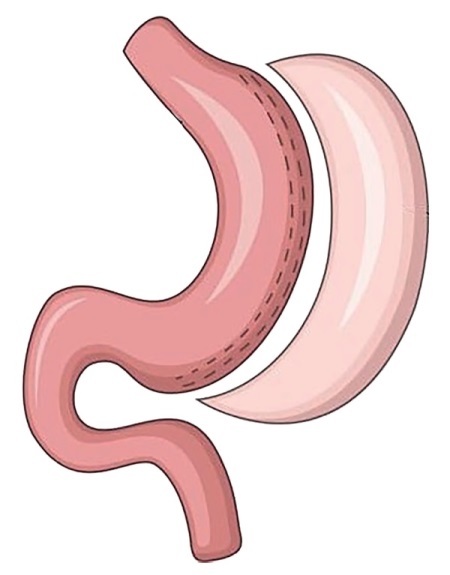
Рисунок 3. Лапароскопическая рукавная резекция желудка.Figure 3. Laparoscopic sleeve gastrectomy

Во время анестезиологического пособия использовался дексаметазон 4 мг внутривенно однократно. В течение 2 ч пациентка находилась в реанимационном отделении, после чего активизирована, переведена в палату стационара и начала пероральный прием жидкостей.

На следующий день больная возобновила прием гормональных препаратов в дооперационных дозах. Выписана в удовлетворительном состоянии на амбулаторное лечение через 48 ч после операции. Послеоперационные рекомендации включали прием ингибиторов протонной помпы (ИПП) — эзомепразол, специализированных мультивитаминных комплексов, препаратов кальция (Кальция цитрат) и железа (Феррум Лек).

Первая плановая консультация через 3 мес после операции (16.06.2022 г.). По результатам обследования выявлен латентный железодефицит — 6,6 мкмоль/л (N 9,0–30,4), недостаточность витамина D — 26 нг/мл (N 30–100). Вес на момент наблюдения составил 87 кг, потеря веса 19 кг, процент потери избыточной массы тела (%EWL) — 42,8%.

Была скорректирована терапия: снижена доза ИПП до 20 мг утром натощак, назначен витамин D по 50 000 ЕД в неделю — 2 мес, затем по 20 000 ЕД в неделю на протяжении месяца; увеличена доза препаратов железа в виде приема через день 100 и 200 мг. На фоне снижения массы тела было принято решение снизить дозу преднизолона, принимаемую вечером, до 2,5 мг, утренняя доза 5 мг и доза Кортинефф 0,1 мг утром оставлены прежние.

При следующем плановом визите через 4 мес (7 мес после операции) вес пациентки составил 74 кг, снижение массы тела 32 кг, %EWL — 72,1% (рис. 4).

**Figure:**
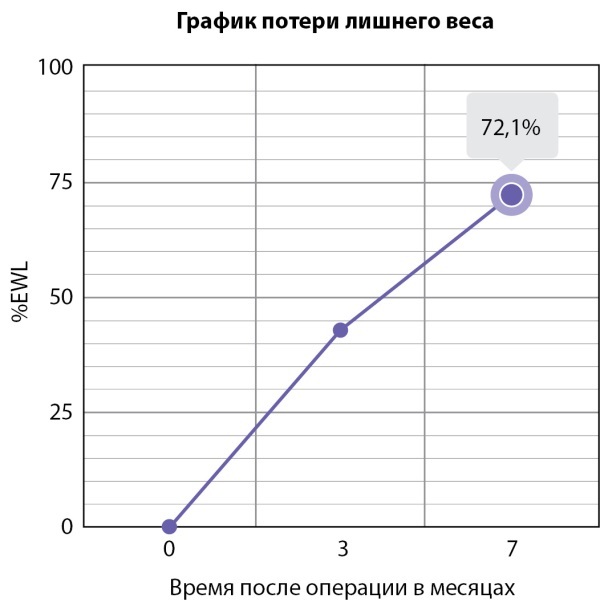
Рисунок 4. График снижения массы тела за 7 месяцев после операции, %EWL.Figure 4. Graph of weight loss for 7 months after surgery, %EWL.

По результатам гормонального исследования все показатели в пределах референсных значений: тестостерон 0,50 нмоль/л (N 0,47–1,7), 17-ОНП 1,58 нг/мл (N 1,24–8,24), ДЭАС 0,82 нг/мл (N<13), андростендион 15 нмоль/л (N 1,6–19), активность ренина плазмы 39,6 мкМЕд/мл, калий 5,2 ммоль/л (N 3,5–5,5), натрий 142 ммоль/л (N 136–145), что говорит о компенсации заболевания. На графиках представлена динамика изменения уровня гормонов крови с 1996 г. (рис. 5.1, 5.2).

**Figure:**
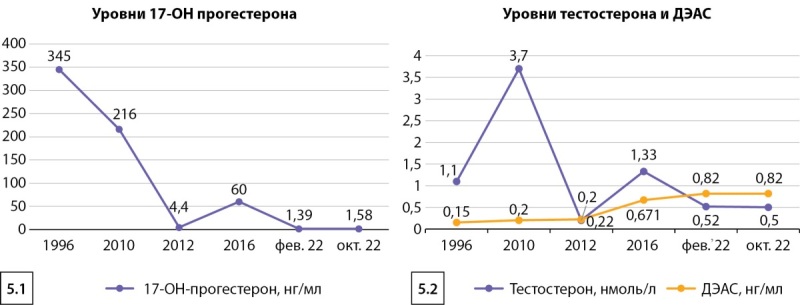
Рисунок 5. Динамика изменений уровней 17-OHП (5.1), тестостерона, ДЭАС (5.2).Figure 5. Dynamics of changes in the levels of 17-OHP (5.1), testosterone, DEAS (5.2).

По результатам биохимического исследования крови показатели в пределах нормы, уровень сывороточного железа на фоне коррекции дозы увеличился до 16 мкмоль/л (N 9,0–30,4), уровень витамина D — до 54 нг/мл (N 30–100). По результатам фиброгастроскопии застойная гастропатия без клинических проявлений, скрининг на Helicobacter pylori отрицательный. Также была проведена двухэнергетическая рентгеновская денситометрия поясничного отдела позвоночника и левого бедра. По результатам исследования бедро-спина наименьшие значения минеральной плотности кости (BMD), измеренные в области левое бедро/все бедро, составляют 0,795 г/см3, что соответствует значению оценки Т-Score=-1,9 и оценки Z-Score=-1,9, а в области спины составляют 0,918 г/см3, что соответствует значению оценки Т-Score=-1,2 и оценки Z-Score=-1,2. Эти данные соответствуют признакам остеопении. По результатам УЗИ органов брюшной полости отмечено уменьшение размеров печени.

Пациентка отмечает улучшение качества жизни после проведенной бариатрической операции на фоне значительного снижения веса. При сравнении результатов опросника SF-36, который пациентка заполнила перед хирургическим лечением, а также спустя 7 мес после, отмечается увеличение показателей по шкалам: физическое функционирование с 40 до 70 баллов, ролевое функционирование с 80 до 100 баллов, интенсивность боли с 43 до 51 балла, общее состояние здоровья с 12 до 15 баллов, жизненная активность с 55 до 60 баллов, психическое здоровье с 35 до 40 баллов. Была скорректирована терапия: ИПП 20 мг, переход на курсовой прием (2 нед прием препарата, 2 мес перерыв), специализированные мультивитамины 1 капсула в день, увеличена доза препаратов кальция до 2000 мг в сутки, доза витамина D по 3000 ЕД в сутки, препараты железа по 100 мг ежедневно.

## ОБСУЖДЕНИЕ

Появление скрининга новорожденных и, как следствие, раннее начало заместительной гормональной терапии у детей с ВДКН позитивно отразились на прогнозе и выживаемости у данной группы пациентов. Ключевым звеном в терапии этой патологии является восполнение дефицита кортизола и альдостерона путем рациональной своевременной коррекции дозировок ГК, что является весьма сложной задачей. Заместительная терапия при этом проводится пожизненно, а ее объем должен быть адекватен факторам, влияющим на фармакокинетику препаратов. И все же имеющиеся в настоящее время лекарственные средства не способны в полной мере воспроизвести физиологическую суточную секрецию. Вследствие этого большинство больных как в детском/подростковом, так и во взрослом возрасте сталкиваются с рядом осложнений терапии ГК. Среди наиболее частых наблюдаются прогрессирующее ожирение и метаболический синдром.

Известно, что метаболический синдром способен влиять на метаболизм кортизола. Показано, что клиренс кортизола обратно пропорционален чувствительности к инсулину, а наличие неалкогольной жировой болезни печени (НАЖБП) также связано с повышенным клиренсом кортизола [10][11]. Исходя из этого, можно сделать вывод, что улучшение чувствительности тканей к инсулину и уменьшение выраженности стеатогепатоза, что происходит при снижении веса, несомненно, позитивно отражается на фармакокинетике гормональных препаратов и снижает пороговые значения минимально эффективной дозы.

Достижение указанных целей может быть реализовано за счет борьбы с ожирением, осложняющим течение основного заболевания. В этом аспекте одним из наиболее эффективным инструментов может выступать бариатрическая хирургия, что подтверждает описанный нами клинический случай. Тем не менее важной особенностью ведения пациентов с данной патологией является опасность возникновения острой надпочечниковой недостаточности в условиях стрессовых ситуаций, к коим, безусловно, относится хирургия. Поэтому разработка и реализация правильной стратегии лечения у пациентов данной группы возможны только благодаря слаженной работе мультидисциплинарной команды — бариатрический хирург, бариатрический эндокринолог, анестезиолог.

С точки зрения выбора типа бариатрического вмешательства у пациентов с ВДКН, на наш взгляд, стоит отдать предпочтение видам операций без гипоабсорбтивного компонента, оптимально — продольной резекции желудка. Ввиду отсутствия достоверных данных по изменению фармакокинетики гормональных препаратов на фоне проведения шунтирующих операций не исключено, что ключевой эффект данных процедур теоретически может усугубить течение основного заболевания из-за увеличения дозы ГК за счет снижения их абсорбции.

Полученный у данной пациентки результат в виде достижения компенсации заболевания, а также снижения дозы ГК демонстрирует влияние сопутствующих заболеваний, а именно МО на тяжесть течения основного заболевания.

## ЗАКЛЮЧЕНИЕ

Представленный клинический случай наглядно демонстрирует возможность выполнения бариатрических вмешательств у пациентов с ВДКН и МО. При правильной периоперационной организации всех этапов лечения с участием бариатрической группы в составе эндокринолога, анестезиолога-реаниматолога, диетолога, хирурга и полноценной подготовке больного соотношение потенциальной пользы и потенциальных рисков бариатрической операции склоняется в выгодную для пациента сторону. Данное наследственное заболевание не должно рассматриваться как противопоказание к проведению бариатрической операции при имеющемся МО, однако выполняться хирургические вмешательства у таких больных должны лишь в крупных бариатрических центрах с максимальным всесторонним опытом в метаболической медицине.

## ДОПОЛНИТЕЛЬНАЯ ИНФОРМАЦИЯ

Источники финансирования. Работа выполнена по инициативе авторов без привлечения финансирования.

Конфликт интересов. Авторы декларируют отсутствие явных и потенциальных конфликтов интересов, связанных с содержанием настоящей статьи.

Участие авторов. Зацепина Е.А., Самойлов В.С. — концепция и дизайн исследования; Зацепина Е.А., Новичихина Е.Е. — сбор и обработка материала; Зацепина Е.А., Новичихина Е.Е., Самойлов В.С., Степаненко А.В. — клинический материал; Зацепина Е.А., Степаненко А.В. — анализ полученных данных, написание текста; Самойлов В.С., Волынкина А.П. — редактирование и финальное утверждение рукописи. Все авторы одобрили финальную версию статьи перед публикацией, выразили согласие нести ответственность за все аспекты работы, подразумевающую надлежащее изучение и решение вопросов, связанных с точностью или добросовестностью любой части работы.

Согласие пациента. Данные пациента деидентифицированы, информированное согласие пациента на публикацию клинического случая получено.
